# Oxidative Stress and Vascular Damage in the Context of Obesity: The Hidden Guest

**DOI:** 10.3390/antiox10030406

**Published:** 2021-03-08

**Authors:** Ernesto Martínez-Martínez, Francisco V. Souza-Neto, Sara Jiménez-González, Victoria Cachofeiro

**Affiliations:** 1 Departamento de Fisiología, Facultad de Medicina, Universidad Complutense de Madrid-Instituto de Investigación Sanitaria Gregorio Marañón (IiSGM), 28040 Madrid, Spain; franvasc@ucm.es (F.V.S.-N.); saraji02@ucm.es (S.J.-G.); 2 Ciber de Enfermedades Cardiovasculares (CIBERCV), Instituto de Salud Carlos III, 28040 Madrid, Spain

**Keywords:** endoplasmic reticulum stress, obesity, oxidative stress, vascular damage, perivascular adipose tissue

## Abstract

The vascular system plays a central role in the transport of cells, oxygen and nutrients between different regions of the body, depending on the needs, as well as of metabolic waste products for their elimination. While the structure of different components of the vascular system varies, these structures, especially those of main arteries and arterioles, can be affected by the presence of different cardiovascular risk factors, including obesity. This vascular remodeling is mainly characterized by a thickening of the media layer as a consequence of changes in smooth muscle cells or excessive fibrosis accumulation. These vascular changes associated with obesity can trigger functional alterations, with endothelial dysfunction and vascular stiffness being especially common features of obese vessels. These changes can also lead to impaired tissue perfusion that may affect multiple tissues and organs. In this review, we focus on the role played by perivascular adipose tissue, the activation of the renin-angiotensin-aldosterone system and endoplasmic reticulum stress in the vascular dysfunction associated with obesity. In addition, the participation of oxidative stress in this vascular damage, which can be produced in the perivascular adipose tissue as well as in other components of the vascular wall, is updated.

## 1. Introduction

The vascular system is comprised of a large number of different vessels that play a central role in the movement of blood throughout the circulatory system. Its main function is the transport of cells, oxygen (O_2_), nutrients and energy between different regions of the body, depending on the needs. In addition, the transport of carbon dioxide (CO_2_) and other metabolic waste products to the exterior (through the lungs and urinary system) is also provided by the vascular system [1].

The function and structure of each component of the vascular system vary depending on the organ it supplies. The structure of blood vessels, aside from capillaries, is composed of three different layers [2]:

- The outer layer, or adventitia, providing structural support and shape to the vessel. The adventitia in the large arteries also supplies oxygen and nutrients to the vascular vessel through the vasa vasorum. This layer is composed mainly by fibroblasts, among other cells [3,4].
- The middle layer or media composed of elastic and muscular tissue which modulates the internal lumen of the vessel. This layer is mainly composed of vascular smooth muscle cells [5].
- The inner layer or intima, composed of endothelial cells that surrounds the interior of the vessel and provides an interface between the blood and vessel wall. These act as sensors for different stimuli, including mechanical (flow and pressure) and/or circulating humoral and inflammatory factors [6].

**Figure 1:**
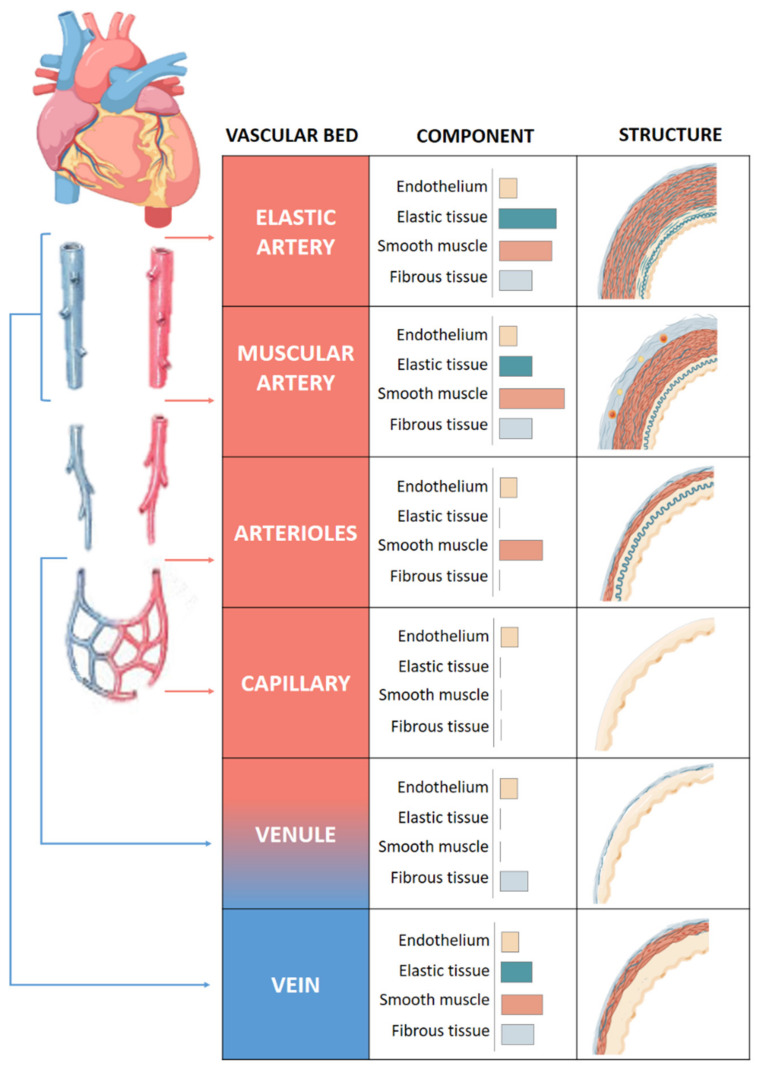
Structure of vascular system. Comparison of the walls of an elastic artery, muscular artery, arteriole, capillary, venule, and vein is shown.

The quantity of muscle and collagen fibrils within each layer varies depending on the size and location of the vessel (Figure 1). Arteries, arterioles and capillaries are the components of the arterial system. Arteries have an abundance of elastic tissue and less smooth muscle due to exposure to high pressure. This high level of elastin allows them to increase in size and modify their diameter, thus conferring to the vessels the elasticity and compliance properties necessary for the correct functioning of the vascular system. Elastic and muscular arteries are the two main types of arteries. The first ones, such as the aorta, contain more elastic tissue and less smooth muscle cells than the muscular arteries. This allows the aorta to maintain a relatively constant pressure gradient despite the constant heart pumping action.

Arterioles that provide blood to the organs contain mainly smooth muscle cells and play an important role in the systemic vascular resistance due to the lack of elastic tissue in the walls. Arteriolar lumen controls the flow of blood into the capillaries, where the exchange of nutrients and metabolites occurs mainly by diffusion [7].

Venules receive blood from capillaries and they can participate in the exchange of oxygen and nutrients [8]. They are the smaller component of venous system with very thin walls prone to rupture with excessive volume. Venules flow into veins composed of three layers like arteries, although less elastic and with a high capacitance that allows it to hold a high volume of blood. They bring the blood toward the heart in a forward direction thanks to the presence of two flap-like structures that regulate blood flow.

The aim of this review is to describe the impact of obesity in this structure and the functional consequences. In addition, the potential mechanisms involved in this damage will be explored with special attention to the roles of perivascular adipose tissue (PVAT), renin-angiotensin-aldosterone system (RAAS) and endoplasmic reticulum (ER) stress. Moreover, the involvement of oxidative stress in these alterations and mechanisms will be discussed.

## 2. Vascular Remodeling in Obesity

Blood vessels respond to mechanical and hemodynamic stimuli associated to a variety of diseases, including hypertension, diabetes and obesity, by modifying their structure, which can result in changes in vessel lumen caliber [9]. Vascular remodeling occurs as an adaptation response to restore wall tension and normalize wall stress in order to maintain the appropriate lumen size for normal blood flow [10].

Vascular remodeling, in general, but specifically in the context of obesity, is an active procedure that involves changes of cell processes at multiple levels, including cell growth, migration, death, cytoskeletal organization, calcification, dedifferentiation of vascular smooth muscle cells (VSMCs) and extracellular matrix (ECM) remodeling. These changes can involve interactions among local growth factors, inflammatory cytokines and vasoactive substances in which the oxidative environment can play a central role [9,11,12,13]. These changes can affect the different vessel layers with functional consequences [14].

Different types of arterial remodeling can be distinguished depending on the underlying pathophysiology:

- Hypertrophic involves thickening of the vascular wall due to cellular hyperplasia and/or hypertrophy or deposition of ECM, which determines an increase in wall-to-lumen ratio. This thickening can be inward or outward.
- Eutrophic involves changes in the diameter of the vessel without changes in the wall-to-lumen ratio.
- Hypotrophic involves thinning of wall and a reduction in wall-to-lumen ratio.

Obesity is associated with vascular remodeling, mainly characterized by media thickening and arterial stiffness not only in conduit arteries such as aorta [15,16], but also in small ones such as mesenteric, renal and coronary arteries [17,18,19,20,21,22]. This remodeling was also observed in subcutaneous small arteries from overweight or obese hypertensive patients, which was accompanied by an increase in fibrosis or a reduction in elasticity [23,24]. This hypertrophy involved different mechanisms, including an ECM remodeling or smooth muscle cell hyperplasia.

VSMCs play a central role in the regulation of vascular tone as well as vessel diameter in order to maintain adequate flow to the tissues [25] thanks to its contractile phenotype, the majority in healthy vessels [14]. However, under certain environmental stimuli or pathological conditions, including obesity [26,27] VSMCs switch to a synthetic phenotype, which is characterized by a high proliferation rate and synthesis of ECM, as well as vasoactive factors [28,29]. This phenotype switching is considered to play a central role in vascular remodeling [14]. Proliferation of VSMCs is a common characteristic reported in the vessels in the context of obesity [18,30] which can participate in media thickening and is facilitated by its synthetic phenotype.

Vascular fibrosis is a common feature associated with obesity [15,30,31,32,33] which results from the accumulation of collagen type I, the main type at vascular level, since no changes in collagen III with more flexibility [34] have been reported [15,18,32]. Fibrosis is a dynamic process that results from the balance of ECM production and degradation. In the case of vascular collagen accumulation in the context of obesity, it seems to be consequence of both processes: an increase in its production [15,18] but also a reduction in its degradation due to an increase in its crosslinking, making it more difficult to degrade. Thus, it is supported by the observation that the administration of an inhibitor of a lysyl oxidase (LOX) reduces fibrosis not only at vascular level but also at the cardiac one in obese animals [35]. LOX is an enzyme involved in the covalent cross-linking of collagen and elastin and is thus responsible for the rigidity and elastic properties of connective tissues [36].

Elastin is an ECM protein that provides resilience and elasticity to the arteries, allowing the aorta to reversibly expand and relax with every cardiac cycle. As opposed to what was observed with collagen, obesity is not associated with changes in elastin levels in the aorta [15,16,18]. The relative levels of collagen and elastin determine biomechanical properties of vessels, and the increase in collagen/elastin ratio observed in different models of obesity therefore leads to increased stiffness. Although no changes in elastin levels is associated with obesity, there has been reported a reduction in fenestra number in the internal elastic lamina in mesenteric arteries from obese mice [18]. This reduction affects vascular mechanical properties, thereby making the vessel stiffer [37,38]. This rigidity is further increased due to an accumulation in fibronectin levels that have also been observed in vessels of obese animals [39,40]. Fibronectin is a major determinant of arterial stiffness and plays a pivotal role in cell matrix interactions [41].

Arterial stiffness is not only observed in models of obesity [18,42,43] but is also a feature found in obese patients independently of age or the presence of metabolic alterations [44,45,46,47]. The increase in the pulse wave velocity (PWV) observed in obese patients results in lower vessel distensibility and compliance. It is worth mentioning that an increase in aortic PWV is an independent factor in predicting fatal and nonfatal cardiovascular events [48,49,50].

Vascular calcification is a complex process [51] that has been considered a sign of aging. However, it has been shown in the last years that this can be present in different pathological situations, including obesity. Obese patients showed an upregulation of different markers of vascular calcification, especially in the presence of diabetes, [52] which can be ameliorated after a weight reduction via bariatric surgery [53]. Vascular calcification has been also reported in obese mice that can be reduced with vitamin E treatment [54,55,56]. Indeed, vascular calcification may be relevant to explaining accelerated vascular aging in the context of obesity and it can contribute to the increase of cardiovascular morbi-mortality by facilitating vascular stiffness.

These vascular changes associated with obesity not only triggered functional alterations but can also lead to impaired tissue perfusion that may affect multiple tissues and organs. These changes can also be produced by capillary rarefaction observed in the obesity that affects almost every organ [57,58,59].

## 3. Endothelial Dysfunction in Obesity

Endothelium is an extremely selective barrier for the permeability of macromolecules while separating blood components from vessel wall matrix and tissues. It is also a highly metabolically active organ with a vital role in the vascular homeostasis [60]. Endothelial cells synthesizes and releases a great variety of substances, including vasoactive substances that regulate vascular tone, blood pressure and local blood flow; substances that participate in coagulation, fibrinolysis and inflammatory and immunological reactions; reactive oxygen species (ROS) and also reactive nitrogen species (RNS), involved in the oxidation and nitrosylation of proteins and lipids and growth factors disturbing cell growth, survival and homeostasis [61,62].

Vascular homeostasis requires maintaining a highly regulated balance between a vasodilator state, often associated with antioxidant, anti-inflammatory and antithrombotic properties, and a vasoconstrictor state, often associated with a prooxidant, proinflammatory as well as prothrombotic state [63]. The endothelium guarantees vascular homeostasis through opposing action of substances with vasodilating properties such as nitric oxide (NO), prostacyclin, and hyperpolarizing factor obtained from endothelium and vasoconstrictors such as endothelin-1, angiotensin II (Ang II) and thromboxane A2 (Table 1) [63,64,65].

The most important compound generated by endothelial cells and influencing vascular homeostasis is NO in healthy conditions. It is produced by the constitutive endothelial enzyme NO synthase (eNOS) under the influence of chemical or mechanical factors. The most characterized among these are the activities of endothelial agonists (e.g., acetylcholine, bradykinin) acting on specific endothelial receptors and shear stress [66]. NO plays a crucial role in the cardiovascular system. The continued generation of NO by eNOS has been associated with a healthy vasculature, while the decrease of NO bioavailability for a long time, due to reduced eNOS activity or the reaction of NO with superoxide anions (O_2_^-^), has been linked with cardiovascular disease [67,68,69].

Endothelial dysfunction is the presence of an altered endothelial phenotype distinguished by reduced bioavailability of NO [70] or a predominant generation of vasoconstrictor and proatherosclerotic substances, prothrombotic and proinflammatory factors, generically called endothelium-derived contracting factors [71].

It is widely known that obesity is an independent risk factor for cardiovascular disease and metabolic disorders. Among these, endothelial dysfunction is one of the earliest vascular alterations observed in obesity, a condition in which endothelial cells change to a pro-atherosclerotic phenotype [71,72]. Numerous studies have seen endothelial dysfunction in different obesity models, both in obese animals induced by genetic manipulation [73,74,75,76], dietary [77,78] or induction of neuroendocrine alterations [79,80]. In obese patients, endothelial dysfunction has been observed along with hyperglycemia, inflammation, and oxidative stress [81]. In a study performed by Apovian et al., the authors have observed by histological examinations that macrophage infiltration in subcutaneous adipose tissue is associated with systemic endothelial dysfunction and insulin resistance in obese patients [82]. In agreement with this study, there has been observed a correlation between circulating tumor necrosis factor alpha (TNFα) levels and endothelial dysfunction in obese patients, showing that inflammation could contribute to vascular dysfunction and is an early onset of endothelial damage in obese patients [83]. In addition, endothelial microparticles have been shown to be upregulated in obesity and are independently correlated with endothelial dysfunction in obese women [84]. The obesity elicits impairment in the endothelium-dependent coronary arteriole dilations in older patients [85]. The endothelial dysfunction has been demonstrated even in adolescents [86] and in children with obesity [87,88]. This altered endothelial function affect not only conduit arteries, such as aorta, but also small arteries including mesenteric, coronary, renal or penile arteries [89,90]. This impaired response to endothelial-dependent vasodilators was accompanied by a reduction in eNOS levels or activity and it was improved in response to exercise or diet supplements [91,92,93]. In a recent study, endothelial dysfunction as well as the wall thickening observed in aorta of mice fed with high-fructose were associated with dysbiosis mainly characterized by a reduction of gut microbiota diversity and a reduction in the abundance of beneficial bacteria [92]. All these data support the complexity of the mechanisms involved in the vascular functional alterations that occur in obesity.

## 4. Mechanisms Involved in Vascular Alterations Associated with Obesity

### 4.1. Perivascular Adipose Tissue

PVAT is the adipose tissue surrounding blood vessels. Most arteries and veins in the body are invested with a layer of PVAT, including the coronaries, aorta, and the microvascular beds of the mesentery, muscle, and kidney [94,95,96].

Until recently, it was considered only to be a passive structural support for the blood vessel [97]. However, evidence from the last decades has led to the wide acceptance of adipose tissue as an important endocrine organ highly metabolically active and produce large numbers of substances, called adipokines, that could affect energy metabolism, insulin sensitivity, inflammatory response, as well as blood flow and the vascular tone in a paracrine or/and endocrine manner [97,98,99,100,101,102,103].

PVAT releases different vasoactive substances, such as adipocyte-derived relaxing factor (ADRF) and PVAT-derived relaxing factor (PDRF) [104,105,106,107], adiponectin [104,108], angiotensin-(1–7) [109,110], hydrogen peroxide (H_2_O_2_) [111], leptin [112] and NO [113], among others. These influence vascular function and are highly important in the regulation of vascular physiology, including vascular tone and endothelial function [99,104,111]. The balance between adipose tissue-derived vasodilator and vasoconstrictor mediators could be extremely important for the maintenance of an appropriate vascular tone. In normal physiological conditions, PVAT has an anticontractile, anti-inflammatory and antioxidant effect [114]. Obesity generates both structural and functional alterations in PVAT [115], increasing PVAT mass [116,117,118,119] and producing changes in the secretory profile of adipokines, resulting in a reduction of expression of vasorelaxation factors and increases in vasoconstrictors [120] and oxidative stress [121], further contributing to a decrease in the anticontractile effect of PVAT [122] and promoting vascular dysfunction [123,124]. The dysregulation in secretion of adipokines by the adipose tissue itself is one of the mechanisms that has linked the increasing fat mass in obesity with cardiovascular comorbidities. This may directly affect the pathogenesis of obesity-related sequelae such as cardiovascular disease [125]. In addition, this PVAT remodeling associated with obesity favors infiltration of immune cells, and upregulation of proinflammatory cytokines [115,120,126], facilitating the generation of a proinflammatory environment. Obese mice induced by high fat diet (HFD) exhibited increased mass, hypertrophied adipocytes and high levels of O_2_^-^ and H_2_O_2_ in PVAT from abdominal or thoracic aorta accompanied by a PVAT dysfunction, with lost anticontractile effect and impaired endothelium-dependent vasodilation [109,118]. In one model of long-time HFD, mesenteric PVAT remodeling was associated with an elevated oxidative stress due to an increase in O_2_^-^ levels associated with increased levels of nicotinamide adenine dinucleotide phosphate (NADPH) oxidases and reduced superoxide dismutase (SOD) activity [123]. H_2_O_2_ might act as a PVAT-derived contractile factor in the setting of obesity [127], which could partly justify the reduction of the anticontractile effect of PVAT. This increase in ROS could involve the activation of the G protein-coupled receptor kinase 2 (GRK2), a serine/threonine kinase, since it is able to stimulate ROS production in a NADPH oxidase-dependent manner in cardiomyocytes [128]. Additionally, the genetic deletion of GRK2 in obese mice prevent the altered endothelial relaxation observed in aorta surrounded by PVAT of wild obese mice. Moreover, no differences in acetylcholine-endothelium-dependent relaxation was observed in control or knockout mice in the absence of PVAT independently of their being fed a control or an obesogenic diet [129].

Similarly, PVAT of gluteal subcutaneous arteries from obese subjects showed not only an increase in depot but also in adipocyte area as compared with that of lean subjects and this hypertrophy was accompanied by a reduction in the dilatory capacity of the vessels, recruitment of macrophages and increase in oxidative stress and inflammation [117].

PVAT not only has a protective effect on vascular tone but also regulates vascular wall structure through the release of factors with anti-inflammatory, antiproliferative and antifibrotic factors [130]. However, PVAT dysfunction that occurs in the context of obesity altered this production and could lead to the development of vascular remodeling. In this line, it has been reported that infiltration of macrophages in PVAT is associated with stenosis of coronary vessels in patients having bypass surgery [131]. In a study involving the Framingham Heart Study Offspring cohort, the volume of the thoracic periaortic fat depots was associated with adiposity, as well as with coronary and abdominal aortic calcification, indicating that aortic PVAT is associated with cardiovascular risk factors [116]. Altogether, it is accepted that maladaptive PVAT remodeling has a critical role in vascular dysfunction in the context of obesity.

### 4.2. Renin-Angiotenisn-Aldosterone System

**Figure 2:**
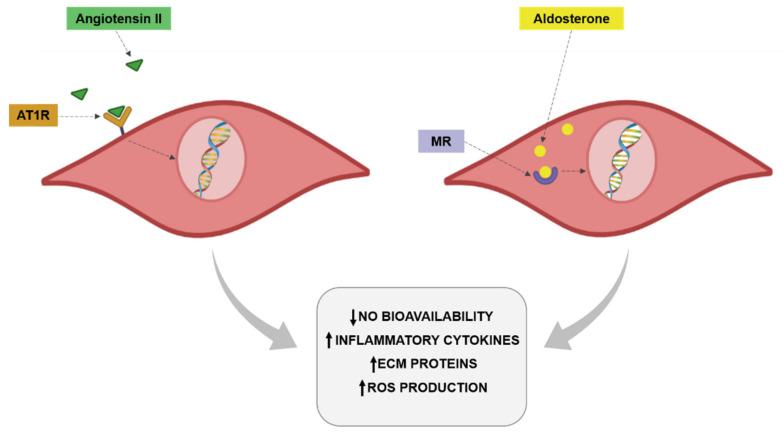
Angiotensin II and aldosterone effects on vascular cells.

The renin-angiotensin-aldosterone system (RAAS) exerts an important impact on the cardiovascular system by participating in the pathogenesis of different pathological scenarios, including hypertension, diabetes and obesity [132]. Obesity promotes increased plasma renin activity plasma angiotensinogen and angiotensin-converting enzyme (ACE) activity, promoting enhanced plasma levels of Ang II in obese patients [133]. It is well established that Ang II is a profibrotic, proinflammatory and prooxidant factor that is also able to induce VSMCs proliferation. Through all these actions, Ang II can participate in the vascular remodeling and the endothelial dysfunction associated with obesity (Figure 2). Indeed, activation of RAAS at a local level or circulating has been found in the context of obesity [134]. Toyama et al. [135] showed a few years ago that the administration of AT1 receptor antagonist telmisartan was able to improve the impaired relaxation to acetylcholine observed in the aorta of a genetic model of obesity in mice. This improvement was accompanied by the normalization of eNOS phosphorylation and a reduction of inflammatory markers. Similar results have been reported in a model of metabolic syndrome in rats not only in aorta but also in coronary arteries. This improvement in endothelial function was again accompanied by amelioration of NO availability [136,137]. In hypertensive obese patients the combination of a calcium and AT1 antagonists was accompanied by improvement of endothelial function and a reduction of inflammatory markers [138]. The reduction of Ang II levels with ACE inhibitors has reported similar improvement in endothelial function in different vascular vessels, including aorta, coronary and epineural arterioles in models of obesity but also in obese patients in which the treatment with ACE inhibitors was able to ameliorate endothelial function in coronary arterioles from obese patients undergoing heart surgery [139,140,141,142]. In hypertensive patients with overweightness or obesity, ACE inhibitor treatment was associated with a reduction in markers of endothelial dysfunction [143].

Regarding the role of Ang II in the vascular remodeling associated with obesity, different studies have demonstrated similar participation to that reported in endothelial dysfunction. The blockade of AT1 receptor was associated with a reduction of coronary artery thickening as well as pericoronary fibrosis in genetic models of obesity [135,144]. Similarly, the inhibition of Ang II was able to reduce the remodeling observed in the abdominal aorta in a model of diet-induced obesity [145].

This improvement was accompanied by a reduction of profibrotic mediators such as transforming growth factor-β (TGF-β) [144]. Clinical studies have reported that inhibition of Ang II improved vascular remodeling of small subcutaneous arteries of obese hypertensive patients and this effect was not observed in those patients treated with a β-blocker despite a similar reduction in blood pressure levels [146]. Blockage of Ang II was also able to improve the media/lumen ratio in subcutaneous small arteries from overweight hypertensive patients and this improvement was associated with a reduction in fibrosis, at least in the patients who received an AT1 receptor antagonist [23]. The combination of an ACE inhibitor with other antihypertensive drugs was able not only to reduce blood pressure levels but also to improve the elastic properties of large arteries in obese hypertensive patients, supporting the beneficial effect of this combined therapy on the organ protection in this kind of patients [24].

It is relevant to mention that clinical and experimental studies have reported that inhibition of RAAS is accompanied by an improvement of the metabolic consequences of obesity [147,148,149,150,151], which can play a relevant role in the vascular consequences in this pathological scenario [152,153]. This beneficial effect has been associated with a reduction in the action/levels of Ang II at vascular level [144,148]. In addition, an activation of the peroxisome proliferator activated receptor-γ(PPAR-γ), a major transcription regulator of multiple genes involved in glucose metabolism, and the participation of Ang (1–7) are other potential mechanisms suggested in this improvement [135,149,151,154].

As already mentioned for Ang II, aldosterone through mineralocorticoid receptor (MR) activation plays a relevant role in the vascular damage associated with different pathological conditions included obesity [155,156,157,158]. In fact, an increase in aldosterone levels in the context of obesity has been also reported [124,156,159,160,161] even in the presence of a high salt-diet [159]. MR are amply found in different tissues, including vessels in which they are expressed in both VSMCs and endothelial cells or perivascular adipocytes [155,156]. An overactivation of MR and excessive aldosterone levels are produced by perivascular adipocytes and can participate in the modulation of vascular function [162,163]. It has been demonstrated that aldosterone exerts prooxidant effects in endothelial cells. This effect is accompanied by a reduction in NO bioavailability (Figure 2). These effects have been confirmed in obesity. Obese mice showed MR activation in endothelial cells, promoting the expression of epithelial sodium channel (ENaC) and oxidative stress along with a decrease in NO and aortic stiffness [164]. Clinical studies have demonstrated that aldosterone plasma levels are increased in obesity and are associated with atherosclerosis progression. Aldosterone is able to increase the expression of intercellular adhesion molecule-1 (ICAM-1) and promotes leukocyte adhesion [165,166]. Aldosterone administration in lean mice, achieving blood aldosterone levels similar to those found in obese mice, promoted endothelial dysfunction in an MR dependent manner. This effect was due to an increase in oxidative stress observed in the animals [167]. It has been described that Rac1, a member of the Rho family of GTPases, is able to increase MR activity. It is important to mention that Rac1 levels is associated with obesity and with oxidative stress [168]. MR activation in VSMCs promotes cell proliferation, migration and calcification, thereby promoting vascular dysfunction and stiffness [169]. The role of MR activation is not limited to VSMCs or endothelial cells. Its activation exerts M1 polarization in resident vascular macrophages, thus enhancing the inflammatory response observed in obesity at vascular level [170].

The role of MR in vascular alterations has been proved by the employment of MR antagonists. Treatment with eplerenone was able to prevent the vasoconstriction induced by aldosterone infusion [171]. In addition, eplerenone prevented the reduced pulse pressure, increased blood pressure levels and vascular stiffness in aldosterone-treated rats [172]. Different studies have shown that treatment with MR antagonists was able to improve endothelial function in diet-induced and genetic models of obesity. In this sense, treatment with the MR antagonism, eplerenone, showed an improvement in vascular reactivity in response to an obesogenic diet. This improvement was accompanied by a reduction in inflammatory cytokines in white adipose tissue, but without any changes in body weight gain induced by the HFD [161,167]. This improvement was also observed in female obese mice [173]. In addition, blockade of MR was accompanied by an improvement in vascular remodeling, perivascular fibrosis, as well as vascular stiffness observed in models of obesity [31,161,173]. This improvement was accompanied by a reduction in ECM components and profibrotic mediators such as TGF-β [161]. Studies in endothelial cell-specific MR knockout mice have reported similar results, showing that the deletion of these improved not only endothelial function but also the vascular remodeling, fibrosis and stiffness observed in obese animals. This improvement was accompanied by a normalization of eNOS levels, reduction of ECM crosslinking and inflammation [164,174]. These observations support the relevant role of the activation of vascular MR in the vascular alterations associated with obesity but specifically at endothelial level. The activation of ENaC in the distal nephron mediated the antinatriuretic effects of the aldosterone [175]. At vascular level, the activation of these channels in the endothelial cells seem to also be the possible mechanism through which aldosterone can mediate the vascular damage in the context of obesity, since specific deletion of endothelial ENaC prevents endothelial stiffness, impaired eNOS activation, aortic fibrosis and remodeling in obese mice through the modulation of vascular oxidative stress and inflammatory response [31]. Finally, it is worth mentioning that the blockade of MR is accompanied by an improvement of the metabolic consequences of obesity [156,176,177,178] and, as we have already mentioned, can participate in the vascular damage in the context of obesity [152,153].

### 4.3. Endoplasmic Reticulum Stress

ER is the cell organelle in which protein synthesis, folding maturation and trafficking take place [179,180,181]. The ER is also responsible for the calcium storage, it being a critical site for the maintenance of cell homeostasis [180,182,183]. Under certain circumstances, the ER is not able to fold the amount of new synthesized proteins, so the unfolded proteins are accumulated in the ER lumen [182], resulting in a state denominated “ER stress”. Under this condition, unfolded protein response (UPR) is activated to restore the ER homeostasis [181,182]. However, a prolonged condition of ER stress leads to the induction of inflammation that results in apoptosis of the unhealthy cells [184]. UPR is a complex signaling network [179,180] which is activated through three different pathways: inositol-requiring protein 1 activation (IRE-1), protein kinase RNA-like ER kinase activation (PERK) and activating transcription factor 6 (ATF6) [179,180]. In normal conditions, these ER membrane-associated proteins are bound by the chaperone GRP78, also called binding immunoglobulin protein (BiP) [180], which keep ER stress sensors inactive. Upon activation of UPR, BiP separates from the three ER proteins, activating the pathways to reestablish ER homeostasis [182] by the degradation of irreversibly unfolded proteins [185].

ER stress is activated by multiple factors such as oxidative stress and calcium overload, or in several pathological conditions such as obesity, diabetes mellitus and cardiovascular disease [183]. Furthermore, ER stress has been suggested as an important mediator in multiple diseases, including cardiovascular or metabolic ones, among others [184,186]. In regard to cardiovascular diseases, ER stress seems to be involved in cardiac remodeling in hypertensive animals, it being an important factor in cardiovascular homeostasis [179]; in ischemic heart disease, lack of oxygen and nutrients due to the ischemic state could impair ER homeostasis and activate UPR, leading to this alteration [187]; in left ventricle samples from autopsy of patients with dilated cardiomyopathy and in mice with hypertrophic and failing hearts induced by transverse aortic constriction, ER stress was activated in myocytes by induction of ER chaperones. Moreover, the administration of an AT1 receptor antagonist reduced ER stress activation, which was accompanied by the prevention of cardiac hypertrophy and failure, as well as a reduction of apoptosis in mice, suggesting that ER stress may be involved in the progression of heart failure [186]. In type 2 diabetes, ER stress also plays an important role due to the fact that a synthesis of insulin occurs in the ER of the pancreatic islet and an increase in insulin production could place a strain on ER function, resulting in an activation of the UPR [180].

Different studies also support the participation of ER stress in vascular pathologies in different diseases. Choi S.K. et al., found in their studies in type 2 diabetic mice that ER stress is responsible for coronary artery dysfunction in these animals, since its inhibition was associated with an impairment of the endothelium-dependent relaxation in coronary arteries [188]. In cardiovascular diseases, ER stress could play an important role in endothelial dysfunction, since treatment with the pharmacological ER stress inhibitors, tauroursodeoxycholic acid (TUDCA) or 4-phenylbutyric acid (PBA), were able to improve vascular reactivity in the animals [189]. Kassan et al., demonstrated in their study that ER stress is a risk factor for vascular alterations in a hypertensive mice model since the treatment with ER stress inhibitors reduces arterial blood pressure and improves endothelium-dependent relaxation, cardiac damage and micro- and macrovascular endothelial function [179]. In another animal model, a hyperglycemic ApoE^-/-^ mouse model, hyperglycemia promotes an increase in ER stress sensors in their aorta walls previous to morphological changes in the vessel structure, showing that ER stress precedes structural and functional alterations and suggests that ER stress possibly exerts an effect on such alterations. [182]. These studies show ER stress as a possible therapeutic target for the vascular alterations associated with different pathologies.

As previously mentioned, ER stress is also induced in obesity and could be a mediator of the development of this pathological context [183,184,186]. ER stress activation has been observed in different situations associated with obesity, such as elevated levels of free fatty acids (FFA) and the following alteration in energy availability [190], insulin resistance and activation of inflammatory pathways [180,181] or accumulation of lipids in ectopic tissues and cells [191].

Some studies suggest that elevated concentrations of FFAs could reduce endothelial NO bioavailability and eNOS activity, leading to obesity-induced endothelial dysfunction [183,190]. These elevated concentrations of FFAs, as mentioned before, could also alter the ER of these cells [190]. ER stress seems to be linked to the activity of eNOS and the production of NO by the endothelial vascular cells, decreasing both of these in this situation [181]. Actually, Lu. et al., demonstrated in a study with rats treated with HFD and fenofibric acid (FF) that HFD induce ER stress (CHOP and BiP levels were elevated) in thoracic aorta, where p-eNOS activity was decreased. Treatment with FF could reduce HFD-induced ER stress and improve p-eNOS activity, resulting in an improvement in NO production. In addition, the authors demonstrated that treatment with the pharmacological inhibitor of ER stress, 4PBA, was able to improve the deleterious effect of palmitic acid (main saturated fatty acid of HFD) on endothelium-dependent vasodilatation, suggesting the involvement of ER stress in the vascular damage associated with obesity [190]. Actually, it has been demonstrated that the inhibition of ER stress in mice treated with angiotensin Ang II was related to an improvement of the endothelium-dependent vascular relaxation that was accompanied by an increase in eNOS activity [181]. The reduction of the activity of eNOS was also demonstrated in coronary artery endothelial cells treated with tunicamycin, another pharmacological ER stressor [181].

Several studies show a relationship between ER stress and oxidative stress in cardiovascular pathologies. It has been described that ER stress induced by tunicamycin was associated with vascular endothelial dysfunction in aorta and other arteries [179,189] through an increase in oxidative stress mediated by NADPH oxidase activity [183]. ER is a site for NADPH oxidase activation, establishing an interaction between ROS and ER stress since ROS is produced during protein folding within the ER and can also induce ER stress [192]. NADPH oxidase is activated by released Ca^2+^ from ER during ER stress [183]. This Ca^2+^ is internalized by mitochondria to generate ROS [181], whose high levels increase oxidative stress and leads to the activation of ER stress and apoptosis, contributing and maintaining endothelial dysfunction [181]. In fact, it has been shown that in high glucose-treated endothelial cells, ROS and ER stress were responsible for apoptosis induction along with a decrease in eNOS expression [183].

The connection between ER and mitochondria is mediated by MAM, the mitochondrial-associated ER membrane [181]. MAM allows the exchange of several compounds, such as Ca^2+^, essential for controlling mitochondrial [193] and cell functions [189], as well as adaptation to pathophysiological conditions which require an enhanced metabolism [182]. Furthermore, this energy demand is enhanced in ER stress situation to control the composition and functions of MAM [181]. The accumulation of Ca^2+^ within mitochondria as consequence of this exchange promotes an enhancement of ROS production and apoptosis [193]. In obese mice fed an HFD or high sucrose diet, where ER stress was induced by tunicamycin, MAM integrity was altered, impairing the interaction between the two organelles. This then results in a Ca^2+^ overload in mitochondria, compromising OXPHOS capacity and augmenting oxidative stress, thereby suggesting that ER stress could be mediating this communication [182,194]. In addition, other studies have found in aorta from mice fed an HFD, both altered MAM and mitochondria, as well as ER morphology in endothelial cells, which were associated with increased ROS production, overexpression of ER stress markers and endothelial dysfunction [182]. The traffic of Ca^2+^ through MAMs is mediated by multiple proteins, including those of BCL family, which promote an increase of this traffic from ER to mitochondria as an adaptive response to the increase of the bioenergetics processes [193].

Several studies suggest that insulin resistance and cardiovascular disease are linked through ER stress in pathologies like obesity; elevated concentrations of saturated fatty acids could impair vasodilatory action of insulin through ER stress in obese individuals [192]. Moreover, it has been described in obese mice that MAM plays an important role in both development and resolution of insulin resistance in hepatocytes, coordinating and mediating ER and mitochondria functions [189].

Thus, obesity-associated ER stress takes place in the development of endothelial dysfunction, since it could initiate and facilitate the maintenance of several pathophysiological states [182,189], although the link to this alteration seems to be unclear.

### 4.4. Central Role of Oxidative Stress in Vascular Alterations Associated with Obesity

Oxidative stress is defined by an imbalance between free radical production and the antioxidant defenses. This imbalance can play an important role in second messengers and intracellular signaling pathways that could affect tissue function. Under this oxidant scenario, free radicals can affect proteins, lipids and DNA, favoring cellular damage and the subsequent tissue injury [195]. There are several sources that can produce ROS at vascular level.

NADPH oxidase (NOX) is a family of enzymes present in the membranes of vascular endothelial cells, VSMCs and fibroblasts [196]. At least seven members have been described in the NOX family [197], which is involved in vascular alterations associated with different pathologies such as atherosclerosis [198,199], Ang II-induced hypertension [200] and diabetes [201], among others. NOX family produces ROS by transferring electrons from NADPH to molecular oxygen [202]. At vascular level, each layer has a particular composition of NOX. Nox4 is expressed by all vascular cells while Nox2 expression predominates in the intima and adventitia layers and Nox1 mainly in VSMCs [203]. Several compounds have been described to activate NOX, such as Ang II via its receptor ATR1 [204], the tyrosine kinase receptor agonist platelet-derived growth factor (PDGF) [205] and TNFα [206]. Obesity is also associated with an activation of NOX [207,208], it being an important contributor of vascular oxidative stress and could drive insulin resistance in this context [209]. At renal level, there has been observed an increase in Nox 1 and Nox 2 in renal arteries, contributing to increased O_2_^-^ production and endothelial dysfunction in obesity [76].

Mitochondria is another important source of free radicals. In the respiratory chain of the mitochondria, ROS are produced and released in function of the superoxide dismutase 2 (SOD2) levels. A decrease in SOD2 levels promotes aortic stiffness by the induction of vascular fibrosis and vascular smooth muscle cell apoptosis in an animal model of aging [210]. Obesity promotes mitochondrial dysfunction and reduced ATP generation in animal models and in patients [211,212]. In addition, it has been described that NOX activation promotes mitochondrial dysfunction and ROS overproduction in an animal model of obesity [213].

Xanthine oxidase acts as another source of free radicals since it donates electrons to O_2_ producing O_2_^-^ and H_2_O2. It is increased in plasma of obese children and is associated with different cardiovascular risk factors such as high-density lipoprotein cholesterol or oxidized low-density lipoprotein [214]. It has been demonstrated that xanthine oxidase and O_2_^-^ production are increased in rat carotid arteries from obese rats and this is associated with alterations in NO endothelium-dependent dilation [215].

All of these sources of free radicals, generate ROS, H_2_O_2_ and RNS among others. It has been described that ROS/RNS, especially H_2_O_2,_ act as second messengers [216]. Second messengers are usually produced in cells after receptor activation; however, some molecules can move from the cell origin acting in a paracrine manner as a second messenger in other cells. In this sense, O_2_^-^ and H_2_O_2_ are generated upon receptor activation and are short-lived, acting as second messengers, and can activate or inhibit signaling pathways, including protein phosphorylation, protein tyrosine phosphatases, protein tyrosine kinases, transcription factors, mitogen-activated protein kinases, and ion channels [217]. These alterations have been proposed to play a critical role in the adipose tissue in obesity. There have been shown the effects of ROS in the development of adipocyte-insulin resistance [218]. In situations in which oxidative stress is exacerbated, ROS are increased and promote adipocyte and mitochondrial dysfunction [219], which could finally lead to adipocyte insulin resistance [220]. These alterations are accompanied by changes in several kinases activity [221], inhibit the proliferation of adipogenic progenitors disturbing adipocytes maturity and inhibit respiration, thereby promoting lipid accumulation [219]. In 3T3-L1 preadipocytes, it has been shown that the exposure to high levels of H_2_O_2_, and in response to glucose oxidation, disrupts the expression of GLUT transporters, leading to a decrease in insulin-stimulated transport of glucose and lipogenesis, and thus to insulin resistance [222]. In addition, high ROS levels could promote an increase in synthesis and secretion of leptin, MCP-1, IL-6 and TNF-α, among others [221]. In addition, ROS exacerbation is associated with lipid accumulation in adipose tissue, adipocyte hypertrophy as consequence of mitochondria dysfunction, adiponectin reduction and the consequent loss of its beneficial effects in insulin resistance, as well as in anti-atherogenic and anti-inflammatory effects [219]. At vascular level, the effects of free radicals as second messengers have been associated with cell growth and migration, regulation of endothelial function decreasing NO bioavailability and the promotion of inflammation and ECM deposition [223].In an animal model of HFD for 6 weeks, we have observed that obese rats presented aortic fibrosis and vascular inflammation even in absence of vascular functional alterations. These structural alterations were accompanied by an increase in O_2_- levels in the aorta of the obese rats [15]. The treatment with an inhibitor of galectin-3 activity was able to prevent all of these alterations [224]. It is important to mention that galectin-3 exerts prooxidant effects in cardiovascular cells despite its profibrotic actions [225]. This interaction between ECM deposition and oxidative stress at vascular level in the context of obesity was confirmed by the use of β-aminopropionitrile (BAPN). BAPN is an inhibitor of LOX activity, which is an enzyme that catalyzes the covalent cross-link of collagen and elastin fibers [36]. HFD animals treated with BAPN for 6 weeks were resistant to developing vascular fibrosis and the increase in ECM proteins. In addition to its antifibrotic effects, the treatment with BAPN was able to prevent the increase in O_2_^-^ production observed in the aorta of the obese animals, as well as in VSMCs treated with leptin, a hormone upregulated in obesity which is involved in the vascular fibrosis observed in obese rats [35]. Obesity induced in young pigs showed impaired coronary endothelium-dependent vasorelaxation and increased oxidative stress characterized by enhanced levels of O_2_^-^, nitrotyrosine and NOX subunits [226].

Cytosolic thioredoxin is an antioxidant which acts as a scavenger of hydroxyl radicals and is also able to restore oxidized proteins and enzymes and induce the antioxidant defense. The lack of thioredoxin generates weight gain in mice and adipose tissue depots accompanied by insulin deficiency in mice. These effects were accompanied by structural remodeling in mesenteric artery characterized by increased wall thickness, hypertrophic remodeling and a decrease in elasticity in the mesenteric artery of the animals fed a HFD and genetically ablation of thioredoxin. All of these alterations could be explained by the enhanced levels of peroxynitrite levels and the subsequent endothelial dysfunction observed in thioredoxin knockout obese mice [227] showing that oxidative stress is associated with vascular remodeling in obesity. The beneficial effects of vascular oxidative stress inhibition were confirmed in transgenic obese mice treated with a chemerin receptor antagonist. This treatment was able to decrease the body weight and vascular insulin dysfunction in mesenteric arteries of the obese animals [228]. In addition, the deletion of MR in endothelial cells reduced oxidative stress, Nox2 expression and renal endothelial stiffness and fibrosis in obese mice [174]. In agreement with this, treatment with a MR antagonist was able to prevent the inward hypertrophic remodeling, the increase number of VSMCs and the vascular stiffness, as well as the increase in Nox 1 and 4 activities and O_2_^-^ production observed in mesenteric arteries from obese animals [229].

The role of oxidative stress in vascular damage has been confirmed by the employment of different strategies such as antioxidants or by the inhibition of different enzymes involved in ROS production. It has been demonstrated that the treatment with a Nox2 inhibitor promoted a prevention of ROS production in aortic PVAT, as well as the subsequent aortic dysfunction in obese rats [230]. As has been mentioned, obesity is associated with alterations in PVAT. In this sense, redox imbalance plays a critical role in the anticontractile effect of PVAT in obesity. Vascular ROS from PVAT in obesity decrease NO bioavailability, it being one of the mechanisms involved in vascular damage in this context. Mice fed a HFD presented an increase in PVAT accompanied by hypertrophic adipocytes in abdominal aorta. These effects were accompanied by an increase in the formation of H_2_O_2_ and O_2_^-^ levels and with impaired endothelium-dependent vasodilation. The presence of the antioxidant enzyme catalase was able to improve the endothelial-dependent vasorelaxation in the aortas in presence of PVAT [118]. In a recent study, Gonzaga N.A. et al., have shown that treatment for two weeks with the antioxidant melatonin was able to restore the anticontractile actions of PVAT, increasing the NO bioavailability in PVAT from rats fed a HFD for 10 weeks. This effect was accompanied by a reduction in O_2_^-^ production in the aorta of the obese animals treated with melatonin and with an increase in the antioxidant defenses in the PVAT of the animals [231]. In agreement with this, another study showed that the treatment with melatonin was also able to restore the anticontractile effects of PVAT from genetically obese mice in mesenteric arteries. This protective effect of melatonin was associated with an improvement in inflammatory markers and with an increase in adipokine with beneficial effects such adiponectin [232]. Melatonin was also able to prevent the prooxidant and profibrotic effects of leptin in the cardiovascular system in the context of obesity [233].

Oxidative stress also mediates the vascular detrimental role of Ang II in obesity. The treatment with tempol, a ROS scavenger, enhanced aorta function in obese mice [234]. There has been described an antioxidant role of vitamin D. Treatment with vitamin D mesenteric arteries from obese patients referred to abdominal surgery was able to improve vascular relaxation capacity, as well as a reduction in ROS production [235]. The antioxidant treatment not only involves improvements in NO bioavailability and the subsequent vascular function. Reduction of oxidative stress by inhibition of xanthine oxidase improves aortic stiffness and vasodilatory responses in obese female mice which were accompanied by a decrease in structural alterations such as aortic fibrosis [236].

These studies show that oxidative stress modulates the endothelial function, as well as the vascular remodeling in the context of obesity.

## 5. Conclusions

**Figure 3:**
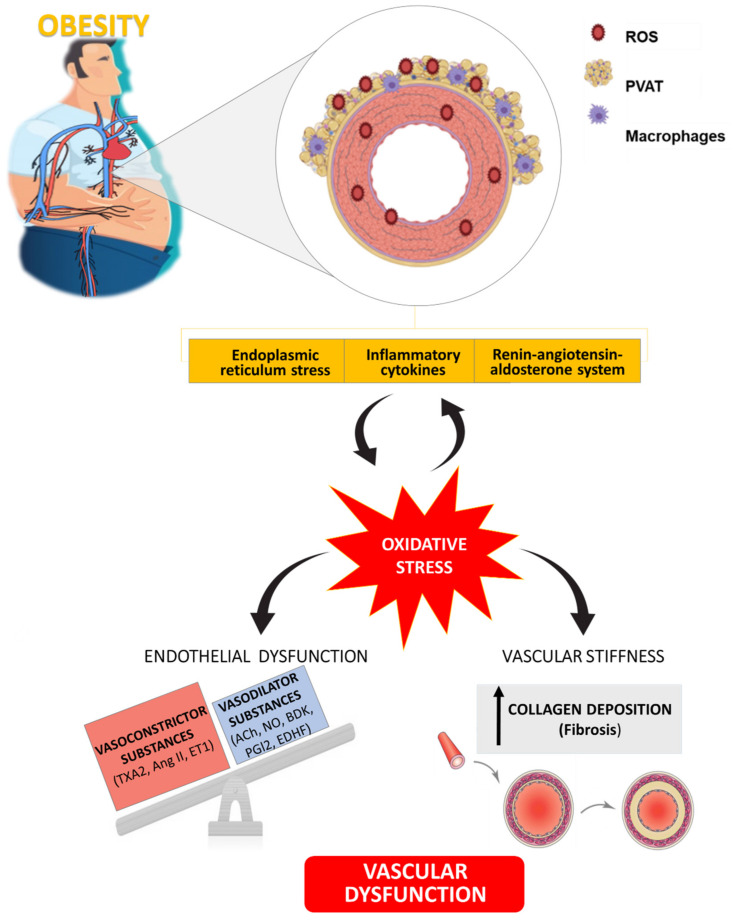
Schematic illustration showing the role of oxidative stress as well as its connection with different mechanisms involved in vascular alterations associated with obesity. TXA_2_: Thromboxane A_2_; Ang II: Angiotensin II; ET1: Endothelin 1; ACh: Acetylcholine; NO: Nitric Oxide; BDK: Bradykinin; PGI_2_: Prostaglandin I_2_; EDHF: Endothelium-Dependent Hyperpolarizing Factor.

This review summarizes the impact of obesity on vascular structure and function and the potential role of RAAS and ER activation or PVAT dysfunction in this damage. In addition, oxidative stress emerges as a downstream process of these mechanisms involved in the vascular dysfunction associated with obesity and highlight its potential benefit as therapeutic target at vascular level (Figure 3). Moreover, a vicious circle could exist between oxidative stress and ER stress, adipose tissue dysfunction and RAAS activation that could be relevant to cardiac damage although less information has been reported regarding the issue.

## Figures and Tables

**Table 1:** Vasoactive factors that regulates vascular tone.

Bioactive Compounds	Effect	Reference
Acetylcholine	Vasodilator	[63,65,66,70,71]
Nitric oxide	Vasodilator	[63,64,65,70,71]
Bradykinin	Vasodilator	[65,66]
Prostacyclin	Vasodilator	[63,64,65]
Endothelium-Derived Hyperpolarizing Factor	Vasodilator	[63,64,65,70]
Endothelin-1	vasoconstrictor	[63,64,65,70]
Thromboxane A2	vasoconstrictor	[63]
Angiotensin II	vasoconstrictor	[63,64,70]
